# Transitions Into Freezing Environments Linked With Shifts in Phylogenetic Integration Between Vitaceae Leaf Traits

**DOI:** 10.1002/ece3.70553

**Published:** 2024-11-14

**Authors:** Charles Tomo Parins‐Fukuchi, Gregory W. Stull, Jun Wen, Jeremy M. Beaulieu

**Affiliations:** ^1^ Department of Ecology and Evolutionary Biology University of Toronto Toronto Ontario Canada; ^2^ Department of Botany National Museum of Natural History, Smithsonian Institution Washington DC USA; ^3^ Department of Biological Sciences University of Arkansas Fayetteville Arkansas USA

**Keywords:** climate, comparative methods, leaf morphology, phylogeny, Vitaceae

## Abstract

Understanding how the intrinsic ability of populations and species to meet shifting selective demands shapes evolutionary patterns over both short and long timescales is a major question in biology. One major axis of evolutionary flexibility can be measured by phenotypic integration and modularity. The strength, scale, and structure of integration may constrain or catalyze evolution in the face of new selective pressures. We analyze a dataset of seven leaf measurements across Vitaceae to examine how correlations in trait divergence are linked to transitions between freezing and nonfreezing habitats. We assess this by applying a custom algorithm to compare the timing of habitat shifts to changes in the structure of evolutionary trait correlation at discrete points along a phylogeny. We also explore these patterns in relation to lineage diversification rates to understand how and whether patterns in the evolvability of complex multivariate phenotypes are linked to higher‐level macroevolutionary dynamics. We found that shifts in the structure, but not the overall strength, of phylogenetic integration of leaves precipitate colonization of freezing climates. Lineages that underwent associated shifts in leaf trait integration and subsequent movement into freezing habitats also displayed lower turnover and higher net diversification, suggesting a link among shifting vectors of selection, internal constraint, and lineage persistence in the face of changing environments.

## Abstract 2

Shifts in the structure of leaf integration correspond to movement between temperate and tropical habitats in the grape clade.

## 1 Introduction

Phenotypic traits often covary. The causes, consequences, and biological significance of trait covariation are complex and manifest distinct patterns across levels of temporal and biological scales. Trait covariation provides a numeric basis for partitioning the phenotype into semi‐autonomous regions, where suites of traits covary internally but are independent of one another. This is referred to as modularity (Wagner, Pavlicev, and Cheverud 2007). The evolution of modularity and its relationship to major unanswered questions in evolutionary theory have long been intuited, but few empirical links have been drawn between the modular patterns that emerge at different levels of the biological hierarchy. Examples at a handful of these levels follow.

Trait covariation has long been used to characterize internal constraints on adaptation within populations of organisms (e.g., Cheverud 1984, 1988; Wagner and Altenberg 1996). At this level, trait covariation is typically thought to reflect the genetic variance/covariance (VCV) matrix, that is, the additive genetic variance shared between each trait pair (Cheverud 1988). The biological significance of this is straightforward. Trait pairs that share a lot of underlying genetic architecture will be constrained in their evolution by the functional demands of each other. The consequences of covariation on adaptation have been fruitfully explored in *Drosophila*. For example, Chenoweth, Rundle, and Blows (2010) found that divergence between nine *Drosophila* populations aligned more closely with the orientation of the VCV than with the direction of experimentally induced sexual selection. In another case study, Hansen et al. (2003) found that the direction and strength of floral evolution over the short term was strongly predicted by constraints induced by covariation. Numerous similar examples exist (Bolstad et al. 2014; Sztepanacz and Houle 2019). However, results are mixed, with many studies suggesting that directional selection can overcome variational constraints (Beldade, Koops, and Brakefield 2002; Agrawal and Stinchcombe 2009). Computer simulations have even shown that directional selection itself may lead to the breakup and rearrangement of patterns in covariation (Melo and Marroig 2015). And so, selection‐induced shifts in the structure of modularity might help facilitate the emergence of new, perhaps complex, adaptations. It appears sensible, then, to conceive of “evolvability”—the ability of a population to respond to selection—as an axis that varies as a function of how well constraints are aligned with selection vectors and the capability for covariation patterns themselves to shift (Houle 1992; Hansen and Houle 2008).

Expanding temporal scale outward, the evolution of covariation patterns has repeatedly come up in paleontological, comparative, and macroevolutionary studies. In these studies, covariation is measured using a diversity of approaches and data sources and so may perhaps be best considered more broadly as reflecting the general structure of modularity that emerges over long evolutionary timescales. It is possible that the origin of new morphologies is facilitated by shifts in the structure of modularity. Qualitative morphological analysis (Vermeij 1973), shifts in patterns displayed by discrete traits (Wagner 2018), and coordinated patterns in evolutionary disparity and rate among suites of quantitative traits (Parins‐Fukuchi 2020a) have all been used to reach this conclusion. Paleontological work also suggests that shifts in the strength of covariation may mediate long‐term trends in phenotypic evolution (Goswami et al. 2015). A parallel but distinct avenue of research has also shown that changes in the strength of correlation between pairs of traits may underlie ecological transitions (Revell and Collar 2009; Revell, Toyama, and Mahler 2022). All these diverse contexts are consistent with the population genetic notion that phenotypic innovations may correspond to changes in “parcellation” and “integration” (i.e., separating and joining together, respectively) of traits (Wagner and Altenberg 1996), but no explicit links have been drawn. The impact of constraints induced by integration patterns on macroevolutionary patterns, such as lineage survival, is also very poorly known.

Trait covariation has also been explored in the context of ecological community assembly. When measured within plant communities, each aligned along an environmental gradient, trait covariation varies as a function of environmental stress (Dwyer and Laughlin 2017; Brown et al. 2022). This pattern probably results from the functional inviability of some trait combinations within certain climates. In this scenario, lineages with unfavorable trait combinations or covariation patterns are filtered out of some regions. While useful from the standpoint of functional ecology, these studies do not tell us how variation in covariation patterns itself arises, nor how or whether shifts in the structure of covariation may underlie the movement of individual lineages into new ecological contexts. Nevertheless, they make it clear that environmental variation plays a major role in patterns of phenotypic integration. This body of work has clearly explained trait covariation in terms of plant ecology; we seek to address it in terms of plant evolution. Our work might complement other recent work bridging plant functional ecology with multivariate patterns in trait evolution (Sanchez‐Martinez et al. 2024).

Here, we perform a novel analysis to identify heterogeneity in the structure of covariation in leaf trait disparity across Vitaceae (grapes and their relatives) to see whether evolvability in multivariate leaf phenotypes coincides with transitions across habitats. Vitaceae represent an excellent system for exploring these problems because it has considerable taxonomic diversity in both tropical and temperate environments, as well as broad variation in leaf form regarding overall size, complexity (simple vs. compound), lobing, and tooth size, structure, and density. Our interests here follow two major themes: (1) identifying whether changes in covariation have the potential to explain major ecological shifts, and (2) reaching across the biological hierarchy to draw more explicit links between the apparently distinct levels of covariation and evolutionary process (microevolutionary, macroevolutionary, and ecological) outlined above. We explored this by applying a novel phylogenetic approach to test for shifts in the structure of covariation in evolutionary divergences across a set of quantitative leaf traits measured across Vitaceae, a clade that has undergone multiple transitions from warm, tropical environments into freezing, temperate biomes. Previous work has found that major changes in leaf phenotype coincide with tropical–temperate transitions in *Viburnum* (Schmerler et al. 2012; Spriggs et al. 2018). We sought to ask whether these changes may themselves be facilitated by rearrangements of the structure of evolutionary covariation among leaf traits. Because leaves possess developmentally integrated suites of traits, it is unrealistic to expect individual traits to respond to climatic changes in simple, predictable ways. Examining changes in the structure of leaf trait integration across climatic shifts offers a basis for understanding the evolutionary underpinnings of environmental transitions, beyond the correlation of individual traits with different climatic variables. As a final goal, we aimed to identify whether the population and quantitative genetic processes that give rise to patterns in the structure of covariation provide any explanatory power over dynamics in phenotypic disparity and lineage diversification across clades.

## 2 Materials and Methods

### 2.1 Data and Code

Leaf measurements across Vitaceae were obtained from Chen (2009). The following seven traits were included: leaf width, leaf length, petiole length, petiole width, distance between secondary veins, tooth location (distance from leaf base), and petiolule length of lateral leaflets (Chen 2009; terminology follows Ellis et al. 2009). For each species, traits were usually measured once from a single specimen (Chen 2009). However, in cases where several measurements were taken, these were averaged, resulting in a single value for each trait for each species. These morphological data were then log transformed prior to analysis. Data files and code associated with this study are available on figshare (https://figshare.com/articles/dataset/vitaceae_data/21754205).

### 2.2 Phylogeny

We used the molecular phylogeny of Vitaceae published by Parins‐Fukuchi (2018). We applied dates to this phylogeny by including the fossil lineages examined in the aforementioned study as node calibrations using treePL (Smith and O'Meara 2012). We did not include the fossils as tips in the dating and covariation analyses because they were known primarily from seeds and therefore would have been uninformative regarding leaf trait covariation. More details regarding the reconstruction of the phylogeny and the full species‐level tree with all tips indicated are included in the supplement (Figure S1).

### 2.3 Covariation in Trait Divergence and “Phylogenetic Integration”

We developed an approach to examine shifts in the structure of evolutionary covariation across a phylogeny. Our intention was to model how the structure of covariation that arises as multiple continuous traits diverge along a phylogeny varies across clades. We term this type of covariation “phylogenetic integration” and use it hereafter. This approach builds upon the work of Parins‐Fukuchi (2020b), by extending the basic framework to explore (1) how covarying evolutionary patterns between traits themselves shift along a phylogeny, and (2) by modeling the covariance structure more explicitly rather than simply relying on shared patterns in phenotypic disparity across traits.

We will start our explanation of the method using the simplest version of the model: one where the structure of covariation is shared across the entire phylogeny. We first perform an ancestral state reconstruction (ASR) under Brownian motion on each of the traits (Maddison 1991). From here, we estimate directional vectors of change along each branch by subtracting the value at each node from that of its parent. At this point, each trait has been transformed into a set of 2n − 2 (*n* is the number of lineages included in the phylogeny) vectors of edgewise evolutionary divergence. We then construct a correlation matrix using the vectors for each trait. This measures the magnitude with which each trait pair undergoes coordinated evolutionary changes and the direction of the association (positive or negative). In other words, it gives the covariance between changes in population means. The precise evolutionary interpretation of this matrix, typically referred to as *V*, has been outlined by Felsenstein (1988) using the equation:

(1) V=GCG

*G* is the genetic covariance matrix, while *C* represents the set of covariances in the selection vectors for each pair of traits. Taken together, *V* is then defined through a combination of the set of genetic constraints and the effects of coordinated selection regimes. Estimating *V* is fundamental to our approach. It provides a natural link between the population genetic conceptualization of covariation and modularity, defined ultimately by *G*, and the patterns observed over deeper timescales, including those explored by paleontologists and macroevolutionists. If we observe shifts in *V*, we are forced to acknowledge the likely reality that those shifts are at least partially facilitated by shifts in *G*. This is because we know, over shorter timescales, that selection tends to be inhibited if it is misaligned with *G*. Of course, the reality is that *C* also likely shifts during major ecological transitions. Any heterogeneity must therefore be considered as the sum of these population processes.

Because estimation of covariance matrices from small datasets can lead to erratic results, we applied the shrinkage approach to estimate the covariance matrices. The result of this procedure is to reduce bias due to high dimensionality and/or low sample size that causes erroneously high and erroneously low covariances. As a result, it should, in principle, help to reduce model overfitting that results from apparent differences in how covariation is structured that result from poorly estimated matrices. Shrunk covariance matrices were estimated using Scikit‐learn (Pedregosa et al. 2011). For this study, we intended to ensure that the minimum clade size entertained would exceed the number of traits in the data matrix and so we defaulted to a mild shrinkage coefficient of 0.1. We placed all traits on the same scale and rescaled all estimated covariance matrices to correlation matrices. Examining covariance matrices instead may also be a useful application of the method, by searching simultaneously for shifts in both evolutionary rate and covariation patterns, but this was not our goal here. To consider the possibility that the structure of modularity has shifted across the phylogeny, we defined a heterogeneous model structured as a phylogenetic mixture of multivariate normal distributions. The likelihood is computed for each branch using the multivariate normal probability density function, with mean vector set to zeros (since net zero change over time *T* is the expectation under Brownian motion) and covariance set to the covariance or correlation matrix computed in the previous step. With the changes for all of the traits along each branch given by the vector *x* and the mean vector given by μ, this yields the expression:

(2) $$L_{\text{branch}}={\left(2\pi \right)}^{-\frac{k}{2}}\det{\left(V\right)}^{-\frac{1}{2}}\;\exp\left(-\frac{1}{2}{\left(x-\mu \right)}^{T}V^{-1}\left(x-\mu \right)\right)$$

The likelihood for the tree is simply the product of the likelihood computed at each *i* of *n* branches:

(3) $$L_{\text{tree}}=\prod_{i=1}^{n}L_{\text{branc}h_{i}}$$

The simplest model assumes *V* is shared across the entire tree. Heterogeneous models allow clades within the tree to possess their own *V*. The likelihood for heterogeneous models is calculated in the same way as for the single *V* model, except that the likelihood for each branch uses the *V* assigned to the clade to which it belongs. To find the best‐supported set of covariance regimes, we employed an automated search algorithm based on that implemented by Smith, Walker‐Hale, and Parins‐Fukuchi (2023). A summary of this algorithm follows.

### 2.4 The Search Algorithm

The algorithm requires a specified minimum size threshold, defined by the number of tips, for clades to be considered. For example, if we specify 10, clades with 10 or more tips will be considered as possible shift points. This greatly improves computational efficiency and helps protect against estimating poorly defined covariance matrices on very small clades. For every clade that meets this size criterion, a covariance matrix is then estimated using *only* the edges subtending the clade root. The algorithm then proceeds as follows: evaluate the likelihood of a combined model, whereby the data are characterized using two multivariate normal distributions, one encompassing the proposed shift and the other encompassing the rest of the taxa in the tree. Calculate the Akaike information criterion (AIC) value using this combined likelihood. If the AIC indicates an improvement in fit, save the estimated parameters and AIC scores; if not, discard them. Rank all the shifts according to their improvement in AIC over the base (single regime) model. Proceed through this ranked list. Retain each model that, when combined with the previously retained models in the ranked list, yields an AIC improvement over the base model. This procedure has the benefit of naturally identifying the optimal shift point in the case where several adjacent nodes all represent possible shift locations. The ranking ensures that the best‐supported location will be added first; others will have to add significantly to the explanatory power of the model if they are to be included as a nested shift. The python code that implements our approach is freely available on GitHub at https://github.com/tomopfuku/multivariate_phylo_shifts. A brief tutorial is also available on the GitHub repository.

### 2.5 Environmental Habit

Movement from tropical into temperate habitats is one of the most significant environmental transitions that plant lineages can undergo. Encountering the freezing temperatures that occur in temperate environments puts substantial physiological stress on plant tissues and often demands specialized adaptations for lineages in these climates to withstand and thrive. We aimed to reconstruct the pattern of evolutionary shifts from nonfreezing to freezing environments in Vitaceae. Hereafter, we will also sometimes use the expression “tropical‐temperate” shift interchangeably, to reflect the tight relationship between freezing tolerance and shifts into temperate climates. First, we generated a dataset of spatial occurrences across Vitaceae by gathering data from the Global Biodiversity Information Facility (GBIF—https://www.gbif.org/). We subjected these raw occurrences to a cleaning procedure using the *CoordinateCleaner* R package (Zizka et al. 2019). We excluded records that matched the location of country capitals and centroids, those with equal latitude and longitude or zeros for both, and any occurrences in the ocean. We acknowledge that a more exhaustive approach, incorporating occurrences from a broader range of databases, including smaller country‐specific ones, might offer certain advantages, particularly for detailed studies of environmental niches. However, we believe that our coarse analysis is not compromised by relying solely on the occurrences available on GBIF, which are already quite extensive. Additionally, we were concerned that merging databases of various sizes and curation practices could introduce redundant information. To avoid biases from including undersampled lineages, we excluded any species with fewer than 10 occurrences after cleaning. We extracted climate data across these locales using the Bioclim dataset (https://www.worldclim.org/data/bioclim.html). We defined any lineage as freezing tolerant for which 2.5% or more of occurrences across their sampled geographic range experience minimum temperatures at or below 0°C during the coldest month of the year. We then performed a parsimony analysis to map freezing tolerance to the phylogeny to compare the location of shifts in covariation structure to those in the environment.

### 2.6 Diversification Rate Analysis

We estimated lineage‐specific diversification rates using MiSSE (see Vasconcelos, O'Meara, and Beaulieu 2022a), a likelihood‐based, hidden state‐only, state speciation, and extinction model implemented in *hisse* (Beaulieu and O'Meara 2016). We set the sampling fraction to be 14.6% to reflect that our tree is an incomplete, but random sample of the total diversity across Vitaceae. Within MiSSE, there are 52 possible models to evaluate, so we used the function *MiSSEGreedy*() to automate the process of fitting a large set of MiSSE models. The function first runs a chunk of models, determines the “best” based on AIC, and then continues on from that complexity until all models in a chunk of complex models are greater than 10 ∆AIC units than the current best model. In this way, we only evaluated a set of models that are reasonably parameter rich with respect to the data set. We culled the resulting model set to remove any redundant model fits. For example, if the maximum‐likelihood estimates are the same for turnover rate—a measure of how rapidly lineages are both going extinct and speciating, calculated as the sum of speciation and extinction rates—in Regime *A* and the turnover in Regime *B* in a turnover rate varying‐only model, it is essentially the same as including a single turnover rate model twice. This would lower the weight of other models as a consequence. It is recommended in these situations to remove the more complex of the two from the set (Burnham and Anderson 2004). For each model, we obtained the marginal reconstructions of the specified hidden states for each node and tip in the tree. We then summarized results based on diversification rates model‐averaged across only the tips that survived to the present. For a given model, the marginal probability of each rate regime is obtained for every tip, and the rates for each regime are averaged together using the marginal probability as a weight: a weighted average of these rates is then obtained across all models using Akaike weights.

We used a paired *t*‐test to assess whether model‐averaged diversification rates are different across the different evolutionary covariation regimes. However, before conducting this analysis we first identified all “cherries” in the tree, which are two tips that are sisters to each other and share the same branch length to the direct ancestral node. Within MiSSE, all sister tips should theoretically inherit the exact rate class probabilities, meaning they have identical tip rates. This could artificially inflate or reduce any means within a given regime. Therefore, as a precaution, we removed, at random, one of the two taxa represented in a cherry (see Vasconcelos, O'Meara, and Beaulieu 2022a).

## 3 Results

### 3.1 Shifts in Evolutionary Integration

**FIGURE 1:**
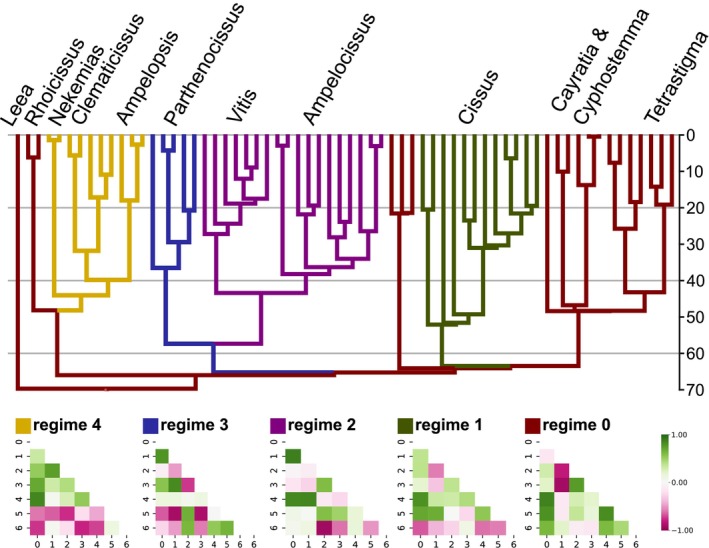
Reconstructed macroevolutionary integration regimes mapped to Vitaceae phylogeny and reconstructions of covariation patterns displayed by each regime. Gradient ranges from −1 correlation (dark violet) to 1 (dark green). Scale lines represent timeline of millions of years.

Our approach uncovered evidence for five distinct evolutionary covariation regimes across Vitaceous leaf traits (Figure 1). The ancestral regime is broadly distributed across the tree, encapsulating *Rhoicissus*, *Cayratia*, *Cyphostemma*, *Tetrastigma*, and several *Cissus* lineages. Our analysis found that the structure of evolutionary covariation shifted at several discrete points throughout the course of Vitaceae's diversification. Our algorithm discovered five covariation regimes in total, each of which displays a distinct correlation structure between the leaf traits. Many trait pairs display different magnitudes in correlation and several also have different signs when compared across regimes. AIC can have the capacity to overfit, but the visible differences in the structure of the shrunk correlation matrix between leaf traits suggest that there likely are real biological differences driving the model heterogeneity. A Newick‐formatted phylogeny file with each internal node labeled with its covariation regime can be found in the file “regimes.tre” packaged within the data supplement on figshare for closer inspection.

### 3.2 Climate Niche Evolution

**FIGURE 2:**
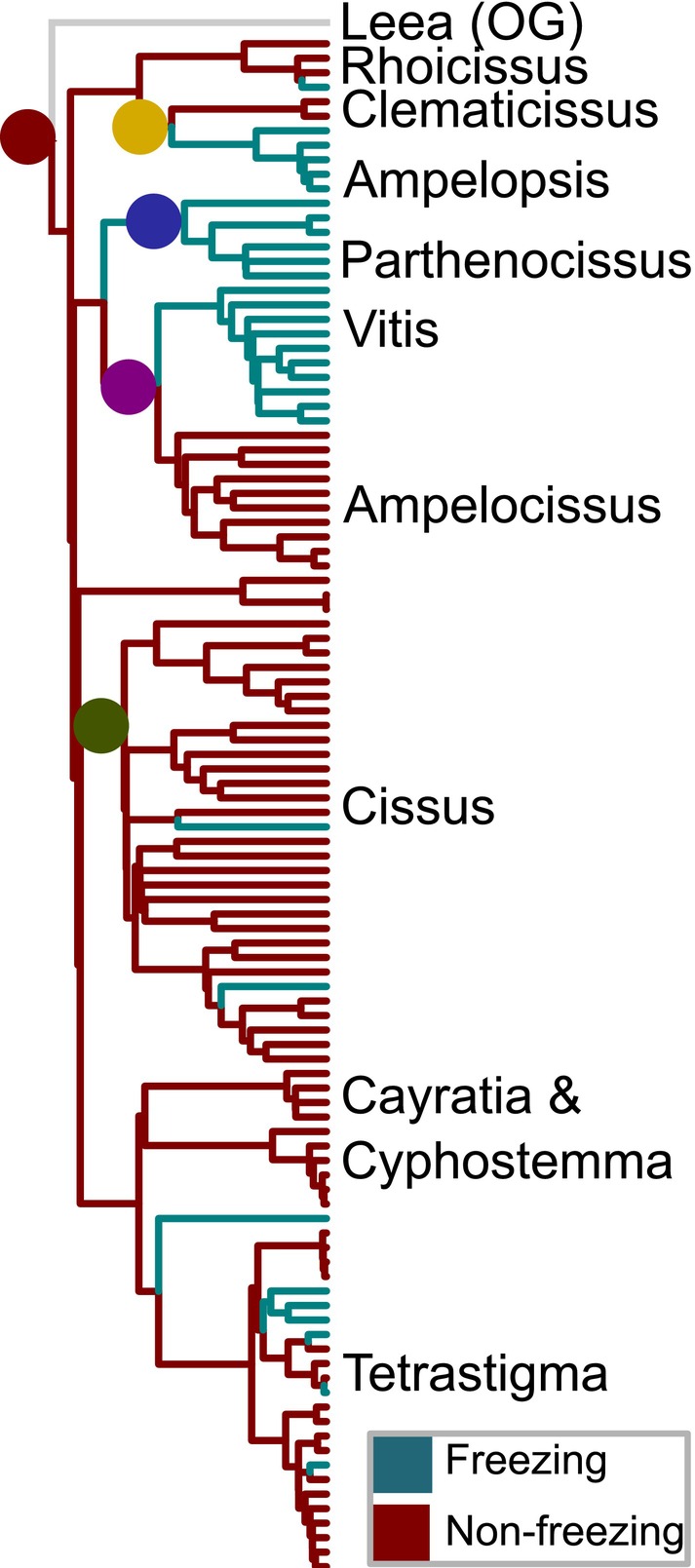
Freezing tolerance mapped to Vitaceae phylogeny. Colored dots correspond to shift points in the structure of phylogenetic integration. Reconstruction of climate tolerance was performed on a superset of the taxa available for the morphological analyses. Taxa not included in the morphological analyses were assumed to follow the same integration pattern as their nearest sibling taxa that were present in the morphological analysis.

Vitaceae displays two distinct modes of climate niche evolution, as it relates to freezing (Figure 2). We reconstructed the most recent common ancestor as nonfreezing. The clade composed of *Cissus*, *Cayratia*, *Cyphostemma*, and *Tetrastigma* was largely nonfreezing. Climate niche was relatively labile in this clade, with several lineages having transitioned into freezing habitats. Despite this flexibility, none of the freezing lineages are very speciose, suggesting that their occupation of freezing regions may be transient. Contrastingly, three Vitaceae clades independently made significant and stable transitions into freezing habitats from warm weather ancestors. The three clades defined by *Vitis*, *Parthenocissus*, and *Ampelopsis*, respectively, each made their own transition into freezing habitats. These lineages maintained their newly derived habits, diversifying only after encountering these new environments.

### 3.3 Habitat Shifts, Integration, and Preadaptation

Several of the shifts in the structure of evolutionary leaf integration across Vitaceae precipitated major movement from nonfreezing habitats into primarily freezing habitats. We observed this associated pattern particularly strongly in Regimes 2, 3, and 4. The emergence of each of these regimes coincides with or immediately precedes the movement of each of these clades into freezing habitats from the tropical ancestor (Figure 2). During this time, global temperatures were considerably warmer than the present, and freezing conditions were perhaps only present (if at all) at high elevations or high latitudes (> 50° N/S; Greenwood and Wing 1995), at least until the onset of climatic deterioration in the mid‐to‐late Eocene (Zachos et al. 2001). Most Cretaceous‐to‐early Cenozoic Vitaceae fossils are known from middle to low latitudes (between 45 N and 45 S; e.g., Chen and Manchester 2007; Manchester, Kapgate, and Wen 2013; Herrera et al. 2024), suggesting that the ancestors of most major lineages likely did not experience freezing conditions. However, early representatives of Vitaceae may have possessed traits (e.g., speculatively and deciduousness) that predisposed them to thrive in freezing conditions. Our analysis suggests that evolutionary shifts in leaf trait integration may have facilitated major environmental shifts in Vitaceae, by emerging earlier, predisposing the lineages encompassed within 2, 3, and 4 to changing environmental conditions, perhaps enabling rapid adaptation and radiation into these new niches. This scenario illustrates how evolution of the phylogenetic integration structure (and by extension, its constituent parts, *G* and *C*) likely facilitated tolerance of unpredictable and distinct habitats, encountered either by migration into new areas or in response to long‐term climate changes occurring in situ.

The sole shift that was not associated with a major tropical–temperate shift was Regime 1, encompassing only *Cissus* lineages. Overall, lineages contained within Regimes 0 and 1 can be characterized as predominantly tropical, with several excursions into temperate space. As a result, it is likely that this shift reflects some other aspect of leaf functional biology. While we focused on tropical–temperate shifts here (with respect to freezing), we also note that the species included in Regimes 0 and 1 also occupy diverse habitats with respect to precipitation, including shifts into xeric environments in continental Africa, Madagascar, and parts of the Neotropics, which might similarly be influencing patterns of trait integration in this regime. It is thus possible that the phenotypic shifts undergone in Regime 1 are functionally related to environmental changes, but future work will be needed to better understand the surrounding context.

We found that the overall correlation strength was fairly uniform across the regimes (Table 1). We found that increased trade‐offs induced by the more stressful climates occupied by lineages diversifying in temperate regions lead to a more generally volatile structure of evolutionary integration. This is highlighted by the repeated shifts in correlation structure in clades that have made decisive shifts into freezing environments. Nevertheless, there remains a key difference at the macroevolutionary scale as compared to ecological scale. While some climates may filter out lineages based on trait covariation patterns over ecological scales, our work shows how lineages themselves can shift the structure of trait covariation and occupy new habitats over long timescales. Thus, instead of observing a weakened correlation structure as lineages radiate into more challenging environments, we found instead that the *structure* underlying those correlations evolves.

**TABLE 1:** Mean overall strength of correlation for each regime.

Covariation regime	Mean absolute correlation strength
0	0.51
1	0.50
2	0.45
3	0.55
4	0.54

### 3.4 Diversification Rates Across Covariation Regimes

We grouped Regimes 0 and 1 into a single diversification regime and compared against rates within a separate grouping of Regimes 2, 3, and 4 to test the hypothesis that the repeated independent movements from tropical into temperate habitats undertaken by Regimes 0 and 1 may have led to distinct macroevolutionary dynamics. We found significant differences in rates of speciation, extinction, net diversification, and turnover when comparing across covariation regimes (Table 2). We found that the lineages that underwent shifts in leaf integration as a precursor to temperate diversification (Regimes 2, 3, and 4) exhibit lower turnover and higher net diversification rates than predominantly tropical lineages making ephemeral movements into freezing habitats (Regimes 0 and 1). The repeated climatic shifts observed across Regimes 0 and 1 correspond to overall higher turnover and marginally lower net diversification. More climatically stable regimes, which are also more tightly integrated, turnover less and generally have a higher net increase in diversity as a result.

**TABLE 2:** Mean diversification rates across covariation regimes.

Covariation regime	*λ*	*μ*	Net diversification (*λ − μ*)	Turnover (*λ + μ*)
0 + 1	0.553 (±0.168)	0.483 (±0.190)	0.045 (±0.009)	1.037 (±0.178)
2 + 3 + 4	0.450 (±0.142)	0.384 (±0.165)	0.058 (±0.006)	0.834 (±0.153)

*Note:* We partitioned the tree into the clade composed of Regimes 0 and 1 and Regimes 2, 3, and 4 because of the distinct patterns in climate niche evolution in each clade and to improve statistical power. In all cases, the differences shown are significant based on a paired *t*‐test (*p* < 0.10).

## 4 Discussion

### 4.1 Ecological and Functional Leaf Morphology and Climate Shifts

A close relationship between leaf form and climate has long been recognized (e.g., Givnish 1987; Spicer et al. 2021). This is reflected by the repeated, independent evolution of particular leaf syndromes and functional traits in similar climates—for example, more rounded, toothed, and lobed leaves in temperate environments (e.g., Schmerler et al. 2012). The widespread use of leaf physiognomy as basis for paleoclimatic inferences is a testament to the close link between leaf form and climate (e.g., Wolfe 1971; Torres Jiménez et al. 2023), but this relationship can be complicated (Peppe et al. 2011). For example, leaf traits concentrated in particular biomes or climatic regimes might be, at least in part, a byproduct of select lineages being overrepresented in those areas (Hinojosa et al. 2011; Little, Kembel, and Wilf 2010). Leaf forms associated with certain climates might also, in some cases, have arisen before the climates themselves, suggesting that new climatic regimes can serve as a filter for preadapted forms (Ackerly 2004).

Over deep time, the functional impacts of trait correlations may differ from those at the ecological scale. Ecological work has found that more stressful environments host plant communities with stronger covariation between functional traits. This is because environmental stressors induce functional constraints that disadvantage certain trait combinations. Lineages that display unfavorable trait combinations are filtered out of certain areas. In this case, trait covariation serves as a catalyst, rather than constraint, for some lineages to move into more challenging environments. Nevertheless, trade‐offs imposed by competing environmental stressors appear to create slightly more complex dynamics in covariation patterns (Brown et al. 2022).

Petiole length plays a major part in the positioning of leaves along an axis, thereby optimizing sun exposure for photosynthesis (Yamada, Suzuki, and Yamakura 1999). Long petioles can also help vines and lianas climb, either via twinning or by simply offering greater reach for the plant to find support (Sperotto et al. 2020). Vitaceae are a clade of lianas, and although tendrils are known to be the primary climbing mechanism of the family (Wen 2007), leaves with elongated petioles may help these plants climb as well. Finally, petiole thickness shows a relationship with leaf construction (thicker petioles are needed for larger or thicker leaves), and generally thicker petioles are believed to offer great mechanical stability for the leaf (Yamada, Suzuki, and Yamakura 1999). The trade‐offs involved in leaf construction—thicker, longer lasting (evergreen) leaves versus thinner, cheaper leaves (deciduous)—are a significant component of plant life history strategies. Therefore, we should expect petiole thickness to be of ecological significance, even if the relationship is indirect. Although functional and physiological significance for several of the traits we examined can be extrapolated clearly, we hesitate to speculate too much about the other traits. Even if particular leaf traits do not have obvious or singular ecological functions, they can nevertheless speak to the extent and patterning of integration among leaf traits as a whole. As a result, all the leaf traits examined here are useful even if their ecological significance is not totally known.

Given that different suites of traits are associated with megathermal (“tropical”) versus mesothermal (“temperate”) climates (Wolfe 1995), we might expect the structure of leaf trait covariance to differ between these types of environments. We also might expect the strength of integration to control the extent to which lineages are able to readily shift between such environments, with more relaxed integration creating an opportunity for more frequent tropical–temperate shifts. The dataset we analyzed here captures different properties of leaf size, venation, and tooth density, traits that have clear relationships with both temperature and precipitation levels (Spicer et al. 2021). Our results broadly show that integration regimes of leaf traits correspond closely with climate, with the strength of integration determining the ease with which lineages can evolutionarily shift into different environments. The coincidence of shifts in phylogenetic integration with movement into novel habitats suggests that leaf traits may function together to facilitate survival in these new environments. In the case of Vitaceae, the shifts in phylogenetic covariation tend to slightly anticipate movement into freezing regions. This mild temporal decoupling might suggest that multiple, perhaps complex, historical circumstances unrelated to the later colonization of freezing climates accumulated in the early evolution of Vitaceae cause shifts in the structure of genetic, selective, and/or environmental correlations. Nevertheless, these “happy accidents” may have prepared Vitaceous clades (especially *Vitis* and *Parthenocissus*) to move into freezing temperate areas and thrive. Functional interactions between combinations of traits often lead to major evolutionary outcomes, such as increased diversification rate, a process that has been termed “synnovation” (Donoghue and Sanderson 2015). While our approach cannot in itself offer the physiological or ecological context needed to make deep biological interpretations, it can perform in an exploratory capacity to identify suites of traits undergoing shifts in the structure of their evolutionary, functional, and developmental interactions. These shifts may then be compared to the broader context (e.g., diversification, environmental niche, and ecological interactions) to determine if they could have yielded major evolutionary consequences.

### 4.2 Leaf Evolvability, Climate Shifts, and Macroevolutionary Dynamics

**FIGURE 3:**
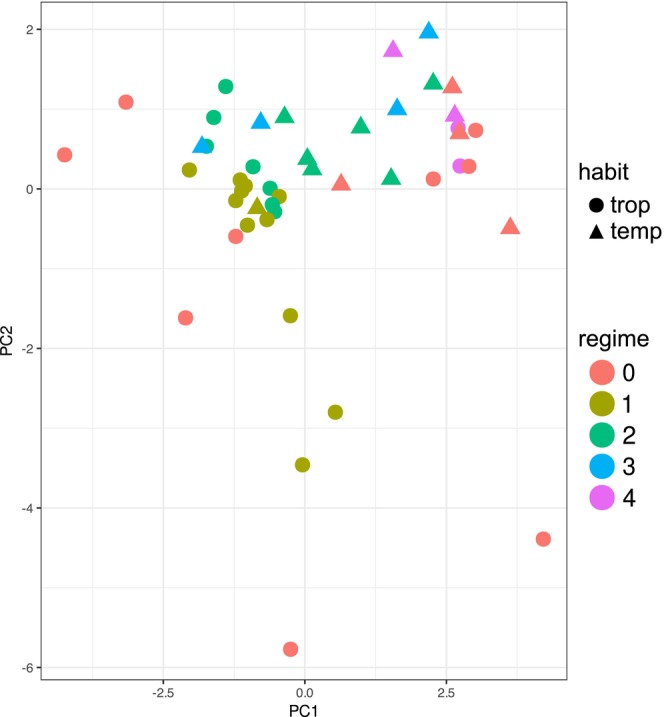
Vitaceae leaf trait morphospace represented using the first two principal components (PCs) calculated from the leaf measurements. PC1 and PC2 explain 52% and 27% of the variance within the dataset, respectively. PC1 is explained by several traits, but aligns most strongly with lateral leaflet petiolule length, while PC2 aligns most strongly with petiole length (Table S1). Species belonging to Regimes 0 and 1 display a much wider spread along both PC1 and PC2 as compared to the rest of the clade. Species belonging to Regimes 0 and 1 have undergone more transitions between freezing and nonfreezing habitats compared to the rest of the tree (Figure 2).

The shifts in phylogenetic integration we observed between leaf traits are the consequence of both shifting structures of multivariate selection and genetic constraints. While it was not possible here, given data limitations, to disentangle the relative influence of each of these in shaping patterns emergent at the phylogenetic scale, the coincidence of major shifts from tropical and into freezing environments with shifts in the structure of trait covariation suggests that vitaceous leaves are generally evolvable in response to environmental changes. Nevertheless, we also observed a complex pattern in leaf morphospace as it relates to climate evolution and evolutionary integration. Regimes 0 and 1 occupy the largest spread of morphospace (Figure 3). In contrast, the lineages that have become more stereotyped as temperate, housed within Regimes 2, 3, and 4, display lower disparity overall. This might suggest that what allowed these lineages to thrive in newly temperate habitats was a structure of evolutionary covariation compatible with the new selection vectors imposed by these environments. This would allow these lineages to thrive while occupying a relatively small portion of morphospace. On the other hand, the frequent ephemeral movements into freezing environments undertaken by the lineages within Regimes 0 and 1 may have stimulated broader diffusion throughout morphospace. This increased disparity may reflect the less specialized nature of these lineages.

Lineages that have colonized and subsequently diversified in newly temperate habitats display quantitatively and qualitatively different macroevolutionary dynamics than those that have remained primarily in the tropics. The integration patterns deployed by Regimes 2, 3, and 4 were derived from the ancestral regime, precipitating shifts into temperate habitats. These lineages became fairly canalized both morphologically and climatically. The lineages contained within Regimes 0 and 1 displayed different climate niche evolution dynamics, repeatedly making the difficult transition from tropical to freezing. This shows that the ancestral regime displayed a high degree of flexibility by both undergoing rearrangement into new, derived integration patterns that facilitated diversification in new habitats and by facilitating the labile climate changes and broad morphospace diffusion displayed by the remaining lineages. We can therefore characterize Vitaceae macroevolutionary dynamics on two complementary axes as reflecting the ability of lineages to: (1) withstand repeated, but ephemeral, shifts into freezing habitats under a static structure of leaf evolutionary integration (Regimes 0 and 1) and (2) modify the structure of leaf evolutionary integration before colonizing and diversifying in new environments (Regimes 2, 3, and 4).

The multivariate vectors of selection on leaf traits likely shift during movement into new climates. The ability of lineages to withstand repeated shifts into freezing habitats suggests that *G* likely does not strongly constrain response of the population means to the new directions imposed by selection in these new environments. If *G* did maintain long‐term constraints across these transitions, these migrant lineages, constrained within a maladaptive phenotypic space relative to their new habitats, would likely go extinct because of a decreased efficacy navigating these new habitats and competition from perhaps better‐adapted species (Van Valen 1973). Although we analyzed these patterns in light of only *abiotic* (environmental) factors, we assume that new environments will also contain different biotic contexts. And regardless of the relative importance of biotic and abiotic factors in driving these macroevolutionary patterns, as originally formulated, the Red Queen accommodates both. We favor this interpretation given that the two likely work synchronously. We therefore assume that the environmental shifts we identified, along with the corresponding shifts in phenotype and development, may indicate changes in both abiotic and biotic factors.

We observed a pattern of high leaf evolvability across environmental transitions paired with elevated turnover rates in lineages transitioning into derived habitats. Diversification rate variation has been explained by many possible dynamics, for example, latitudinal correlates with energy input: “the Red Queen runs faster when she is hot” (Brown 2014). Our analyses reflect a somewhat simpler dynamic that unifies several patterns across scales. The patterns reconstructed in Vitaceae suggest that *the Red Queen runs faster when she is uncomfortable* (see similar arguments in Stebbins 1974; Vasconcelos, O'Meara, and Beaulieu 2022b). Repeatedly encountering new habitats due to migration and/or climate change results in the emergence of a completely new set of biotic and abiotic conditions that may yield a variety of responses that are intrinsic to each individual lineage. These responses may be rooted in developmental and genetic constraints on phenotypes, population‐level variation, and extinction dynamics. As a result, while extinction tends to increase when lineages encounter new habitats, this is compensated for by increased speciation among phenotypically flexible lineages.

Vitaceae has navigated these dynamics in two contrasting ways. The lineages in Regimes 2, 3, and 4 may have thrived and diversified in new, freezing, habitats by inheriting a modified structure of phylogenetic integration. The lineages in Regimes 0 and 1 appear to withstand repeated encounters with freezing habitats by increasing their macroevolutionary pace, as measured by elevated turnover rates. It is possible that these lineages are more likely to go extinct when they encounter freezing habitats than lineages within Regimes 2, 3, and 4, but can persist over long timescales by maintaining elevated speciation rates. Regimes 2, 3, and 4, which have experienced diversification events in freezing habitats, are thus perhaps more “comfortable” in these new environments due to their derived patterns in genetic and selective constraints. Nevertheless, there may be some morphological bet‐hedging happening. While Regimes 3 and 4 are predominantly freezing and Regimes 0 and 1 are predominantly nonfreezing, Regime 2 (*Vitis–Ampelocissus*) is evenly balanced between both. We found that Regime 2 was the least integrated lineage overall (Table 1), raising the possibility that this simultaneous diversification in both freezing and nonfreezing areas may have been facilitated by an overall more flexible structure of genetic and functional constraint. This would distinguish the macroevolutionary strategy of Regime 2 from Regimes 3 and 4, which appear to be more specialized and canalized in freezing habitats. These three regimes are further distinguished from Regimes 0 and 1 in that they may persist in freezing environments due to possessing modified morphological structures, as opposed to increased rates of speciation. More work mapping the links among phenotypic innovation, constraint, and speciation rates in different lineages will help to further refine our understanding of how lineages persist in the face of a shifting evolutionary landscape.

This second layer might explain results in vertebrates that conflict with latitudinal explanations for diversification rate variation (Rabosky et al. 2018), which found “paradoxically” higher speciation rates far from the tropics. These patterns may reflect a more extreme manifestation of the causes we outline here. Movement to more extreme environments may, in some lineages, increase variation in macroevolutionary parameters (lineage diversification, the origin of phenotypic novelties, etc.) to a level that overwhelms latitudinal patterns. For example, certain ecological conditions in temperate and arctic regions may be so far from a lineage's initial capability to accommodate them that it must increase its macroevolutionary activity beyond that displayed in the tropics to outpace extinction. This may manifest itself in higher turnover, faster and wider phenotypic disparification, and trait correlation patterns that are structured more flexibly. The relative importance of latitudinal versus intrinsic explanations likely varies across clades, environments, and epochs. Deeper understanding of the level(s) at which selection operates and how intrinsic evolvability interacts with movement into new ecological contexts will help to further disentangle the root causes of these dynamics and disparity in patterns across studies and taxa.

It also seems worthwhile to note that the lineages within Regime 0 do not cluster according to climate niche in leaf morphospace (Figure 3). This suggests that, during repeated transitions back into freezing climates, each lineage tends to carve out a unique evolutionary path and ultimately approach similar environmental challenges with different phenotypic solutions. Alternatively, it is possible that variation in other climatic variables is causing these lineages to diffuse into different regions of morphospace. Shifts into arid habitats, which became more widespread during the Neogene, might have influenced leaf evolution and morphospace occupancy in various ways, independently or alongside shifts into freezing conditions.

### 4.3 Caveats

Despite our optimism for our analyses, we acknowledge that several important limitations exist. Felsenstein (1988) gave an explicit critique of the method through which we construct *V*. Specifically, he pointed out that ASRs are not true data, but instead inferences drawn from data. While we, of course, agree on a basic level, we believe the method is sufficient for our aim of reconstructing broad shifts in evolutionary covariation. Practically speaking, while ASRs are often rife with error, we believe that our own questions can be adequately tackled with estimates containing relatively high errors. The most important aspect is identifying positive and negative correlations that particularly stand out and how that structure changes across a tree. It is also worth noting that many phylogenetic comparative methods have arisen since the time of Felsenstein's writing that uses essentially the same information we use here—traits mapped to a phylogeny—to derive historical inferences. We are therefore confident in our approach given our aim, which fundamentally was to present an exploratory analysis. Detailed examination of each pairwise trait relationship, or a full breakdown and interpretation of the *G* and *C* components, may benefit from more careful methodological consideration, or at least further validation that the resulting covariances are numerically robust to this approach. Such detailed investigation will undoubtedly require more sophisticated methods and far more extensive data collected at much higher sample sizes. We are therefore content in this work to present our results as a “first‐pass” from which further, more deeply mechanistic studies can be later derived.

While we introduce our estimation of heterogeneous *V* matrices as a bridge between phylogenetic patterns in trait divergence and population‐level patterns in quantitative genetics, it is also important to note that this link relies explicitly on the conjecture that the phenotypic covariance matrix (*P*) can be adequately substituted for the genetic correlation (*G*) matrix (Cheverud 1988). The statistical robustness of this assumption has often survived scrutiny across a variety of study systems (e.g., Roff 1995; Dochtermann 2011; Sodini et al. 2018), including plants (Waitt and Levin 1998). Nevertheless, even if *P* strongly predicts *G*, it still does so with statistical noise. The strength of this noise will vary in relation to factors such as the specific details of the underlying genetic architecture and the orientation and stability of environmental effects on the phenotype. Over the course of millions of years, these effects could fluctuate considerably. While *V* provides an explicit link between divergence over long timescales and the process of genetic constraint, it is also shaped by other forces so it is important to interpret our results with these assumptions and limitations in mind. For example, in principle, it is possible that the shifts in *V* identified here are the consequence of a reworking of internal genetic architecture, changes to how multivariate selection operates in new environments, or discrete differences in the structure of environmental effects. These three possibilities are probably not equally likely, but our model and the data we examined here are not able to distinguish between them on a statistical level.

### 4.4 Shifts in Phylogenetic Integration as a Scale‐Unifying Construct

Our results provide one illustration of the potential for a hierarchically integrated view of biological modularity. The formulation of our model provides a bridge between quantitative and population genetics, macroevolutionary patterns in multivariate trait disparity and lineage diversification, and ecological dynamics. The modularity that emerges from covariation patterns at each level may combine in complex ways to yield the evolutionary behaviors observed at subsequently higher levels. Phylogenetic integration patterns provide a bridge between these scales and a route through which to more carefully dissect how processes at each scale interact to form the patterns we observe across the tree of life. More broadly, investigating shifts in phylogenetic integration can generate a more hierarchically cohesive understanding of phenotypic evolution. Examining shifts in integration between evolutionary divergences affords the potential to link the cumulative effects of well‐characterized population processes over deep time. This framework can be further leveraged to explain how shifts in multivariate complexity mapped to long timescales correspond to major ecological shifts, thereby making the initial steps in a new framework through which to seek a truly cohesive view of biological complexity across temporal, taxonomic, and spatial scales.

## Author Contributions

**Charles Tomo Parins‐Fukuchi:** conceptualization (lead), data curation (lead), formal analysis (lead), funding acquisition (lead), investigation (lead), methodology (lead), project administration (lead), software (lead), writing – original draft (lead). **Gregory W. Stull:** formal analysis (supporting), investigation (supporting), visualization (supporting), writing – review and editing (supporting). **Jun Wen:** investigation (supporting), writing – review and editing (supporting). **Jeremy M. Beaulieu:** formal analysis (supporting), investigation (supporting), writing – review and editing (supporting).

## Conflicts of Interest

The authors declare no conflicts of interest.

## Supporting information


**Table S1.** Correlations of each of the first three principal components with the original measurements. Larger absolute values indicate that a measurement is more strongly correlated with a principal component.
**Figure S1.** Dated phylogeny of 126 species of Vitaceae used in the study.

## Data Availability

Raw data files and code used to perform the analyses are available at https://figshare.com/articles/dataset/vitaceae_data/21754205. Python code implementing the phylogenetic method is available on GitHub along with a tutorial at https://github.com/tomopfuku/multivariate_phylo_shifts.
