# Supplementary material for: Transitions Into Freezing Environments Linked With Shifts in Phylogenetic Integration Between Vitaceae Leaf Traits
Source: Ecol Evol. 2024 Nov 14;14(11):e70553. doi: 10.1002/ece3.70553 (PMC11563691; doi:10.1002/ece3.70553)
Supplement: Supplementary file 1 — Table S1. Correlations of each of the first three principal components with the original measurements. Larger absolute values indicate that a measurement is more strongly correlated with a principal component. Figure S1. Dated phylogeny of 126 species of Vitaceae used in the study. [file ECE3-14-e70553-s001.docx]

**Supplementary information**

*Phylogeny.* We borrowed the molecular phylogeny of 138 Vitaceae species published by Parins-Fukuchi (2018), which was inferred from eight nuclear and chloroplast gene regions mined from Genbank. We pruned 12 lineages from this original phylogeny that were lacking leaf measurement data, leaving us with 126 species (Fig. S1). For the freezing habitat analysis, 13 species lacked sufficient occurrence information and so we pruned them from the tree as well, leaving us with a phylogeny of 113 species.

| Trait | PC1 | PC2 | PC3 |
| --- | --- | --- | --- |
| leaf_size_width | -0.19496745 | 0.092473031 | 0.4115808 |
| petiole_length | -0.29145938 | 0.921962359 | -0.2377452 |
| distance_between_2_secondary_vein | -0.06032011 | -0.007706685 | 0.3139638 |
| teeth_location_distance_from_base | -0.01249452 | 0.053819358 | 0.3970426 |
| leaf_size_length | -0.19680302 | 0.127985364 | 0.6331487 |
| petiole_width | -0.14348383 | 0.018541393 | 0.2630697 |
| lateral_leaflet_petiolule_length | -0.90217818 | -0.348932937 | -0.2185852 |

**Table S1.** Correlations of each of the first three principal components with the original measurements. Larger absolute values indicate that a measurement is more strongly correlated with a principal component.


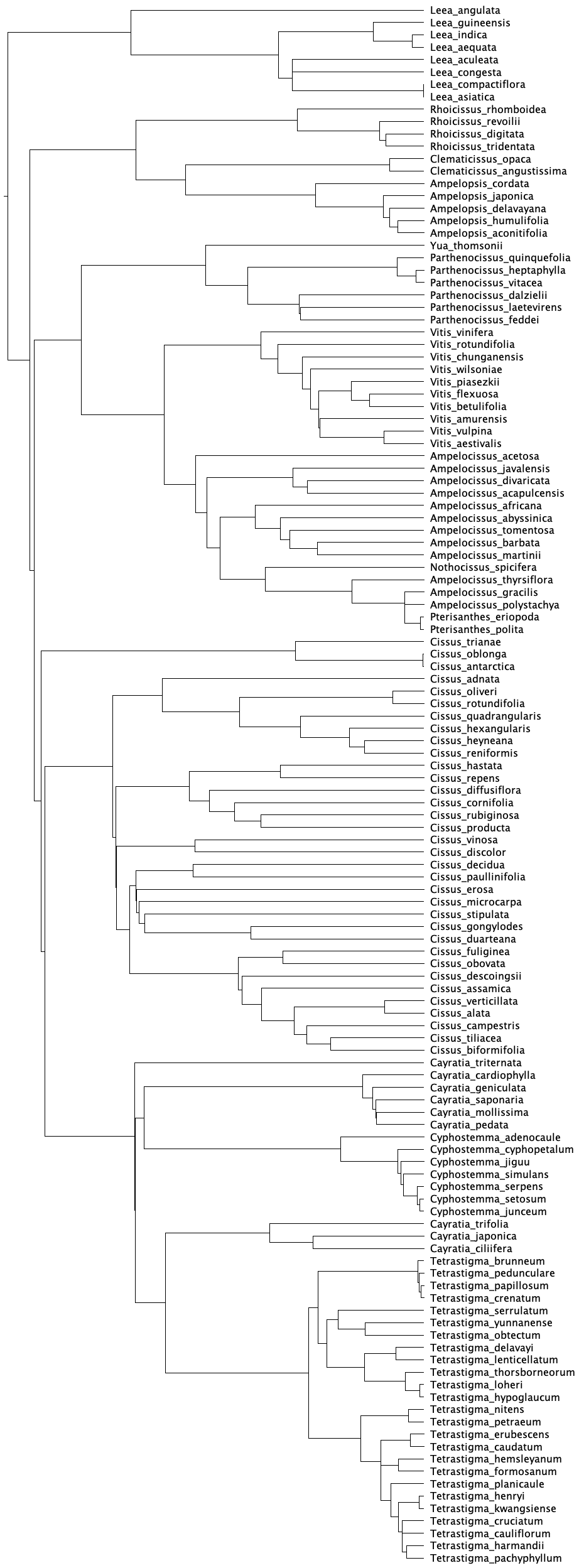


**Figure S1.** Dated phylogeny of 126 species of Vitaceae used in the study.
